# Stability of centrosome numbers in *poly(ADP-ribose) polymerase-1-* and *poly(ADP-ribose) glycohydrolase*-deficient mouse ES cells

**Published:** 2004-06-01

**Authors:** Hideki Ogino, Akemi Gunji, Nobuo Kamada, Hitoshi Nakagama, Takashi Sugimura, Mitsuko Masutani

**Affiliations:** *) Biochemistry Division, National Cancer Center Research Institute, 1-1 Tsukiji 5-chome, Chuo-ku, Tokyo 104-0045, Japan; **) Inst., Chugai Pharmaceutical Co. Ltd., Gotemba, 1-135, Komakado, Gotemba, Shizuoka 412-0038, Japan

**Keywords:** Poly(ADP-ribose) polymerase-1, poly(ADP-ribose) glycohydrolase, centrosome, embryonic stem cell, DNA damage

## Abstract

Poly(ADP-ribose) polymerase-1 (Parp-1) localizes mainly in the nucleus and functions in DNA repair, genome stability and cell death regulation. Meanwhile, it also localizes in centrosomes and is involved in the regulation of centrosome duplication. An abnormal increase in centrosome numbers is frequently observed in *Parp-1*-deficient (*Parp-1*^−/−^) mouse embryonic fibroblasts (MEFs) (Kanai *et al*. (2003) Mol. Cell. Biol. **23**, 2451–2462). However, there are no studies on whether the centrosome abnormality occurs also in other cell types under *Parp-1* deficiency. In this study, we report that *Parp-1*^−/−^ mouse embryonic stem (ES) cell lines did not show an abnormally increased number of centrosomes compared to wild-type ES cells. Recently, poly(ADP-ribose) glycohydrolase (Parg) has also been shown to localize in centrosomes (Ohashi *et al*. (2003) Biochem. Biophys. Res. Commun. **307**, 915–921). The number of centrosomes of *Parg*-deficient (*Parg*^−/−^) ES cells was also analyzed in this study and was found to be stable under *Parg* deficiency. We also examined centrosome numbers in wild-type, *Parp-1*^−/−^ and *Parg*^−/−^ ES cell lines after treatment with methylmethanesulfonate (MMS) or *γ*-irradiation. Although a slight increase in the number of centrosomes is observed in each genotype twenty-four hours after treatment with MMS at 50 μM or with *γ*-irradiation at 1.4 Gy, there was no difference among the genotypes. These results suggest that loss of *Parp-1* and *Parg* is insufficient to induce abnormality in centrosome numbers in ES cells and that ES cells possibly possess a strict mechanism for the maintenance of a normal number of centrosomes.

## Introduction

Poly(ADP-ribose) polymerase-1 (Parp-1) is an enzyme catalyzing polyADP-ribosylation, a post-translational modification.1),2) Parp-1 has a dominant role in polyADP-ribosylation in cells, modifying Parp-1 itself and various other proteins associated with chromatin after being activated by DNA strand breaks. Poly(ADP-ribose) is degraded into ADP-ribose by poly(ADP-ribose) glycohydrolase (Parg) by splitting *α*(1″ → 2′) glycosidic linkages.3)–5) Poly(ADP-ribose) metabolism plays a role in many biological functions including DNA repair,6) cell cycle regulation,7) chromatin remodeling,8) transcriptional activation and repression,9) and protein degradation.10)

Recent studies showed that Parp-1 localizes both in nuclei and centrosomes throughout cell cycles.11),12) An abnormal increase in the number of centrosomes is frequently observed in 3-aminobezamide-treated mouse embryonic fibroblasts (MEFs) and *Parp-1*^−/−^ MEFs,12) and it is shown that replication of chromosomes and duplication of centrosomes are uncoupled in *Parp-1*^−/−^ MEFs.12) Parp-1 also polyADP-ribosylates centrosomal p5312) and may thus modulate its function in centrosomal regulation.

We previously reported that immortalized *Parp-1*^−/−^ MEFs show enhanced ploidy increase compared to wild-type (*Parp-1*^+/+^) MEFs.13) It could be speculated that an increase in centrosome numbers and ploidy coordinately occurred under *Parp-1* deficiency. On the other hand, we reported that *Parp-1*^−/−^ ES cells did not manifest ploidy increase.14) To clarify the relationship of ploidy increase and abnormality in centrosome numbers and to understand the impact of *Parp-1* deficiency on centrosome numbers in cells other than MEFs, we analyzed the number of centrosomes in *Parp-1*^−/−^ ES cells. Since Parg was recently reported to be localized also in centrosomes,15) we analyzed the number of centrosomes in *Parg*^−/−^ ES cells as well.

## Materials and methods

### Cell culture

ES cell lines, J1 (*Parp-1*^+/+^, *Parg*^+/+^), 210–58 (*Parp-1*^−/−^), 226–47 (*Parp-1*^−/−^),14) B609 (*Parg*^+/−^), D79 (*Parg*^−/−^), and D122 (*Parg*^−/−^)16) were maintained in the absence of a STO cell feeder layer in Dulbecco’s modified Eagle’s medium (Gibco) containing 20% fetal calf serum supplemented with non-essential amino acids and leukemia inhibitory factor (ESGRO, Chemicon) as described elsewhere.14) For measurement of centrosomes by indirect immunofluorescence, 10^6^ cells were plated onto 100-mm diameter gelatin-coated dishes (Iwaki). The next day, cells were mock-treated, treated with methylmethanesulfonate (MMS, Sigma) at 50 μM or *γ*-irradiated at 1.4 Gy using ^60^Co. Twenty-four hours after the treatment, cells were fixed for centrosome immunostaining.

### Indirect immunofluorescence

Indirect immunofluorescence was performed according to the previous reports11),12) with slight modifications. Cells were first cooled on ice for approximately 10 min, and then were extracted on ice with cold extraction buffer (pH 6.7 at 25 °C) containing 0.75% Triton X-100, 5 mM piperazine-1,4-bis(2-ethanesulfonic acid, and 2 mM ethylene glycolbis(*β*-aminoethyl ether) *N*, *N*, *N′*, *N′*-tetraacetic acid for 2 min, and fixed with 10% formalin and 10% methanol for 20 min at room temperature. All of the subsequent processes were carried out at room temperature. After washing with phosphate-buffered saline (PBS), cells were permeabilized with 1% NP-40 in PBS for 5 min, incubated with blocking solution (2% bovine serum albumin in PBS) for 1 hour and probed with anti-*γ*-tubulin polyclonal antibody (T3559, Sigma) for 1 hour. The antibody-antigen complexes were visualized with Alexa Fluor^®^ 594-conjugated goat anti-rabbit IgG antibody (Molecular Probes) by incubation for one hour. Cells were counterstained with 4′,6-diamidino-2-phenylindole (DAPI) and were analyzed with a fluorescence microscope (Axiovert 200, Zeiss).

### Statistical test

The significance of differences was analyzed by the Mann-Whitney *U* test and the Kruskal-Wallis test using SPSS version 6.1 software (SPSS Inc.).

## Results

**Fig. 1:**
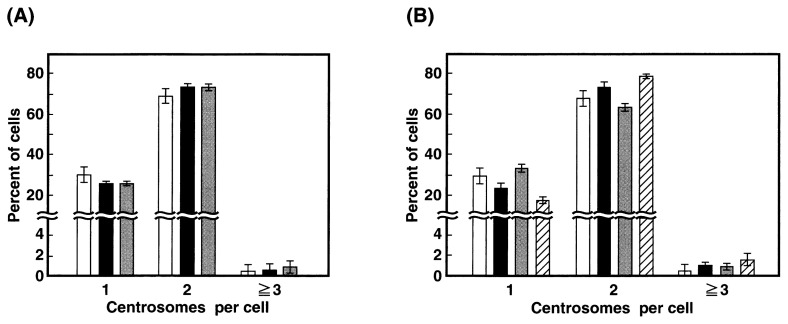
Number of centrosomes in (A) *Parp-1*^−/−^ or (B) *Parg*^−/−^ ES cells in normal culture conditions. Triplicate measurements were carried out. In each measurement, 200–300 cells were counted for centrosome numbers. In (A), white, black, and gray columns represent J1 (wild-type), 210–58 (*Parp-1*^−/−^), and 226–47 (*Parp-1*^−/−^) ES cells, respectively. In (B), white, black, gray, and hatched columns represent J1 (wild-type), B609 (*Parg*^+/−^), D79 (*Parg*^−/−^), and D122 (*Parg*^−/−^) ES cells, respectively. Cells containing no centrosome were not detected in every genotype. Bars indicate mean ± S.D.

The number of centrosomes was counted in an asynchronous culture of *Parp-1*^+/+^ and *Parp-1*^−/−^ ES cells after staining with centrosomal *γ*-tubulin. The result is shown in Fig. 1A. In every genotype, one or two *γ*-tubulin signals were observed in more than 98% of total cells, and there was no difference in the percentage of the cells containing three or more centrosomes between *Parp-1*^+/+^ and *Parp-1*^−/−^ ES cells.

Next, to elucidate whether degradation of poly(ADP-ribose) by Parg is important for centrosome function, we examined centrosome numbers in *Parg*^−/−^ ES cell lines, in which total poly(ADP-ribose) degradation activity was reduced approximately by half. We observed a 3-fold accumulation of poly(ADP-ribose) in these *Parg*^−/−^ ES cells without any treatment compared to *Parg*^+/+^ ES cells (Fujihara *et al*., submitted). An asynchronous culture of *Parg*^−/−^ ES cells was used for the measurement of centrosome numbers (Fig. 1B). Most of *Parg*^+/+^, *Parg*^+/−^ and *Parg*^−/−^ ES cells contained either one or two centrosomes per cell and there was no significant difference among *Parg* genotypes, although D122 cells showed a slightly higher frequency of three or more centrosomes per cell than in *Parg*^+/+^ ES cells (*p* = 0.0495 in Mann-Whitney *U* test). These results indicate that loss of *Parp-1* and *Parg* is insufficient to induce abnormality in the number of centrosomes in ES cells in normal culture conditions.

It is reported that various genotoxic stresses, including treatment with hydroxyurea,17),18) aphidicolin,12) and *γ*-irradiation,19) induce an abnormal increase of centrosome numbers in various cell lines. Therefore, we speculated that an abnormal increase in centrosome numbers might be induced in ES cells after treatment with genotoxic stresses. Wild-type, *Parp-1*^−/−^ and *Parg*^−/−^ ES cell lines were treated with methylmethanesulfonate (MMS) or *γ*-irradiation. MMS treatment was carried out at a concentration of 50 μM, which causes an approximately 2-fold elevation in the frequency of sister-chromatid exchanges16) and *γ*-irradiation was performed at 1.4 Gy. Both treatments did not affect the survival of *Parp-1*^−/−^, *Parg*^−/−^ and wild-type ES cells (data not shown). The results are shown in Table I. After treatment with MMS at 50 μM or *γ*-irradiation at 1.4 Gy, the percentages of cells containing abnormal numbers of centrosomes, namely 3 or more, showed a tendency to slight increase in every genotype. However, there was no significant difference in the increase of centrosome numbers in *Parp-1*^−/−^ and *Parg*^−/−^ ES cells compared to wild-type ES cells.

## Discussion

In this study, we demonstrated that *Parp-1* and *Parg* deficiency did not induce an abnormal increase in centrosome numbers in non-treated conditions and after treatment with DNA damaging agents. The results with *Parp-1*^−/−^ ES cells obtained in this study are in clear contrast with those obtained with *Parp-1*^−/−^ MEFs, with which the number of centrosomes significantly increased compared to wild-type MEFs.12) Since *Parp-1*^−/−^ MEFs showed enhanced ploidy increase13) whereas *Parp-1*^−/−^ ES cells did not show it,14) one possible explanation could be that the ploidy increase may be closely related to the abnormal elevation of the number of centrosomes.

It is reported that percentage of the cells containing three or more centrosomes per cell elevated from 2% to 30% by *Parp-1* deficiency in primary cultures of MEFs.12) We observed that in wild-type, *Parp-1*^−/−^ and *Parg*^−/−^ ES cells, the percentage of cells containing three or more centrosomes was maintained at the level of less than approximately 2%, even after treatment with DNA damaging agents (Fig. 1 and Table I). ES cells may thus possess a strict mechanism to maintain a normal number of centrosomes. Since Parp-320) and tankyrase21) also localize in centrosomes, these Parp family proteins or other proteins may compensate the Parp-1 function in ES cells. It is also possible that ES cells may not be able to survive under centrosome abnormality, and cells with an abnormal number of centrosomes may be immediately excluded by cell death. In *Drosophila* embryos, DNA replication defects and DNA damage trigger the inactivation and disruption of centrosomes depending on the function of checkpoint kinase 2 and this appears to maintain genome stability by blocking chromosome segregation and elimination of defective nuclei.22),23) We did not observe the elevation of the frequency of cells lacking centrosomes in every genotype, even after treatment with DNA damaging agents (Table I), suggesting the possibility that ES cells may not have the pathway of centrosome inactivation and disruption. A detailed time course analysis of centrosome numbers in ES cells after treatment with DNA damaging agents may further elucidate the maintenance mechanism of centrosome numbers in ES cells.

The present results indicate that the impact of *Parp-1* deficiency is substantially different depending on the cell types and *Parp-1* deficiency did not induce the abnormal centrosome numbers in ES cells. This could support the evidence that *Parp-1*^−/−^ mice develop and grow normally without showing extensive genomic instability.

The function of Parg in centrosomes has not been clarified yet, but the presence of polyADP-ribosylated proteins including p53 and Parp-1 itself suggests the requirement of Parg activity for poly(ADP-ribose) degradation in centrosomes. As we presented in this study, *Parg* deficiency did not induce the abnormal increase of centrosome numbers in ES cells. This may be due to the residual activity of Parg present in *Parg*^−/−^ ES cells (Fujihara *et al*., submitted) used in this study. Further study using potent Parg inhibitors or knocking down Parg activity completely with other gene-targeting constructs or with small interference RNA techniques may be helpful for understanding the function of Parg in centrosome regulation.

## Figures and Tables

**Table I:** Percentage of ES cells containing an abnormal number of centrosomes following MMS treatment or *γ*-irradiation

ES cell line	Treatment^b^	Centrosome number per cell^a^					

		3	4	5	6	7	≥ 8
J1 (Wild-type)	Mock-treated	0.5 ± 0.3	0	0	0	0	0
	MMS^c^	0.9 ± 0.3	0.8 ± 0.1	0	0	0	0
	IR^d^	1.9 ± 0.9	0.5 ± 0.2	0	0	0	0.1 ± 0.1

210–58 (*Parp-1*^−/−^)	Mock-treated	0.6 ± 0.3	0	0	0	0	0
	MMS	1.2 ± 0.5	0.5 ± 0.1	0.3 ± 0.1	0	0	0
	IR	0.8 ± 0.3	0.7 ± 0.4	0	0	0	0

226–47 (*Parp-1*^−/−^)	Mock-treated	0.9 ± 0.5	0	0.1 ± 0.1	0	0	0
	MMS	0.9 ± 0.6	1.1 ± 0.6	0	0	0	0
	IR^e^	1.1 ± 0.4	0.7 ± 0.1	0.1 ± 0.1	0	0	0

D79 (*Parg*^−/−^)	Mock-treated	0.6 ± 0.3	0.2 ± 0.2	0	0	0	0
	MMS	0.3 ± 0.2	0	0	0	0	0
	IR	0.1 ± 0.1	0.1 ± 0.1	0	0	0	0

D122 (*Parg*^−/−^)	Mock-treated	1.5 ± 0.2	0.1 ± 0.1	0	0	0	0
	MMS	2.3 ± 0.3	0.4 ± 0.4	0	0	0	0
	IR	1.2 ± 0.2	0.1 ± 0.1	0	0	0	0

a Measurements were done in triplicate. In each measurement, 200–300 cells were counted for centrosome numbers. Cells which contained no centrosome were 0.2 ± 0.2^c^, 0.3 ± 0.1^d^, 0.3 ± 0.1^e^%, respectively, whereas other samples contained at least one centrosome. Values (%) represent mean ± S.E.

b MMS treatment at 50 μM and *γ*-irradiation (IR) at 1.4 Gy were performed as described in **Materials and methods** and cells were fixed 24 hrs after each treatment.
